# Commentary: Strategies to Address COVID-19 Vaccine Hesitancy and Mitigate Health Disparities in Minority Populations

**DOI:** 10.3389/fpubh.2021.723583

**Published:** 2021-07-29

**Authors:** Jason Teo

**Affiliations:** Faculty of Computing and Informatics, Universiti Malaysia Sabah, Kota Kinabalu, Malaysia

**Keywords:** COVID - 19, machine learning, vaccines, datasets, adverse reactions

## 1. Introduction

This commentary builds upon the article recently published by Strully et al.(1), which highlights vaccine hesitancy particularly among minority groups and proposes strategies to mitigate this serious setback in combating the pandemic. In light of the surging third wave of COVID-19 due to more virulent variants, the aim of this commentary is to generate further discussion and debate on the urgent need to focus efforts in making available datasets for prediction of adverse reactions from COVID-19 vaccinations. The goal of achieving herd immunity is at the moment hampered by anti-vaxxers and general skepticism from the unvaccinated arising from concerns regarding the safety of COVID-19 vaccines. With a publicly-available COVID-19 vaccine adverse reaction dataset, the machine learning community would be able to conduct predictive analytics studies and develop prediction tools to allay fears of adverse reactions based on the individual health backgrounds of the potential vaccine candidates, and this could arguably increase the confidence of the unvaccinated to get vaccinated.

As of 11 June 2021, more than 175 million cases of COVID-19 affecting 219 countries and territories have resulted in more than 3.7 million deaths (Worldometer Report). This COVID-19 outbreak, caused by the severe acute respiratory syndrome coronavirus 2 (SARS-CoV-2), was first identified in Wuhan (Hubei, China) in December 2019 and has since swept across the entire globe, turning COVID-19 into a full-blown global pandemic by March 2020 (WHO), which is expected to cause up to $2 trillion in global GDP losses (UN News Report).

As countries scramble to secure vaccines to inoculate their citizens as a means of ending this pandemic, reports of adverse events arising from the urgent rush to mass vaccinate are being reported across the globe. As reported in the Business Insider (2), the Guardian (3), and CNN (4) on 12, 14, and 15 March 2021, respectively, a number of reports from major news sites have highlighted that more than a dozen countries have now suspended the use of a particular brand of COVID-19 vaccine due to severe adverse events. As such, rapid identification of patterns and trends relating to COVID-19 vaccine adverse events is beyond crucial, particularly from the use of computer-based simulations and modelling. In this vein, though data scientists are keen to assist, the large majority are sidelined due to the lack of detailed COVID-19 vaccine adverse event datasets. Although a handful of datasets related to COVID-19 vaccine adverse events are available, none of them contain any useful patient-level data such as reported symptoms and/or medical histories of the patients or are highly geographically-constrained in terms of their data source (5). If such datasets with detailed patient information are made available, a number of critically needed machine-learnable tasks would immediately become possible:

1. Prediction of susceptibility to adverse events based on health attributes such as physiological indicators, bloodwork, and/or medical history;
2. Prediction of the likelihood of fatalities due to adverse events;
3. Prediction of short-term versus long-term forms of adverse events that require hospitalization;
4. Prediction of adverse events probabilities across different vaccine brands for high-risk patients.

Apart from contributing to COVID-19 vaccination buy-in, such a system would also be beneficial to global vaccination programs against other vaccine-preventable infectious diseases. As highlighted by Salman (6), there is still a very significant shortfall in vaccine adverse events monitoring and reporting worldwide. Although improving COVID-19 vaccination acceptance is currently a crucial global agenda, the application of machine learning to the analysis and potential reduction/prevention of adverse effects of susceptible groups of vaccine recipients will remain a critical agenda for all routinely-administered vaccines. In order to materialize such a machine learning approach for vaccine safety monitoring, some potential ways forward may include:

- A globally-accessible central vaccine adverse events data repository with high quality datasets that are maintained and curated professionally [see for example (7)];
- A standardized and homogeneous data format for reporting adverse events [see for example (8)];
- Automated scheduled analysis and reporting of adverse events *via* machine learning approaches [see for example (9)];
- Incentives and competition-based data mining of adverse events by the machine learning community [see for example (10)].

Hence, detailed patient-level data related to COVID-19 vaccine adverse events that are rapidly made openly available is key for the application machine learning to the safe roll-out of COVID-19 vaccinations globally. The solution would need the concerted efforts of all nations to agree to contribute their patient data to a central vaccine adverse events repository (managed by the WHO?) in a timely manner that is made accessible to the public in real-time. This should perhaps become of the main global goals in order to bring the COVID-19 pandemic under control through safer mass vaccinations augmented by machine learning. Importantly, the advancements made in the application of machine learning to COVID-19 vaccine safety monitoring would be highly relevant and easily extended to other routinely administered vaccines for the same purpose. Therefore, these efforts would not only have a temporary contribution in overcoming the current pandemic but will continue to benefit the global vaccination program against other infectious diseases well beyond COVID-19.

## Author Contributions

The author confirms being the sole contributor of this work and has approved it for publication.

## Conflict of Interest

The author declares that the research was conducted in the absence of any commercial or financial relationships that could be construed as a potential conflict of interest.

## Publisher's Note

All claims expressed in this article are solely those of the authors and do not necessarily represent those of their affiliated organizations, or those of the publisher, the editors and the reviewers. Any product that may be evaluated in this article, or claim that may be made by its manufacturer, is not guaranteed or endorsed by the publisher.

## References

[1] Strully KW Harrison TM Pardo TA Carleo-Evangelist J. Strategies to address COVID-19 vaccine hesitancy and mitigate health disparities in minority populations. Front Public Health. (2021) 9:645268. 10.3389/fpubh.2021.645268 33968884 PMC8102721

[2] Dean G Schuster-Bruce C. These 5 Countries Have All Suspended AstraZeneca's Vaccine Over Possible Side Effects while 6 Others Have Banned a Specific Batch of Shots (2021). Available online at: https://www.businessinsider.com/astrazeneca-covid-vaccine-countries-suspend-denmark-thailand-batch-blood-clots-2021-3 (accessed March 15, 2021).

[3] Boseley S. Netherlands Joins Ireland in Vaccine Suspension Over Blood Clot Concerns (2021). Available online at: https://www.theguardian.com/world/2021/mar/14/ireland-suspends-oxford-astrazeneca-covid-vaccine-over-blood-clot-concerns (accessed March 15, 2021).

[4] Picheta R. Spain, Germany, France and Italy Pause AstraZeneca Vaccine Rollout (2021) Available online at: https://edition.cnn.com/2021/03/15/europe/italy-lockdown-europe-coronavirus-monday-scli-intl/index.html (accessed March, 16 2021).

[5] U S Department of Health and Human Services (HHS). VAERS Data (2021). Available online at: https://vaers.hhs.gov/data.html (accessed March 15, 2021).

[6] Salman O. Progress in immunization safety monitoring worldwide, 2010–2019. MMWR Morbid. Mortal. Wkly. Rep. (2021) 70:547–51. 33857066 10.15585/mmwr.mm7015a2 PMC8344995

[7] OpenNeuro. A Free and Open Platform for Sharing MRI, MEG, EEG, iEEG, ECoG, ASL, and PET Data (2021). Available online at: https://openneuro.org/

[8] PRIDE. Proteomics Identification Database (2021). Available online at: https://www.ebi.ac.uk/pride/markdownpage/pridesubmissiontool (accessed June 25, 2021).

[9] Hicks SA Eskeland S Lux M de Lange T Randel KR Jeppsson M. Mimir: an automatic reporting and reasoning system for deep learning based analysis in the medical domain. In: Proceedings of the 9th ACM Multimedia Systems Conference. Amsterdam. (2018). p. 369–74.

[10] Kaggle. SIIM-FISABIO-RSNA COVID-19 Detection (2021). Available online at: https://www.kaggle.com/c/siim-covid19-detection (accessed June 25, 2021).

